# *GmBZL3* acts as a major BR signaling regulator through crosstalk with multiple pathways in *Glycine max*

**DOI:** 10.1186/s12870-019-1677-2

**Published:** 2019-02-22

**Authors:** Li Song, Wei Chen, Biao Wang, Qiu-Ming Yao, Babu Valliyodan, Ming-Yi Bai, Ming-Zhe Zhao, Heng Ye, Zhi-Yong Wang, Henry T. Nguyen

**Affiliations:** 1 Joint International Research Laboratory of Agriculture and Agri-Product Safety, Jiangsu Key Laboratory of Crop Genomics and Molecular Breeding, Co-Innovation Center for Modern Production Technology of Grain Crops, Yangzhou University, Yangzhou, 225009 China; 2 Division of Plant Sciences, University of Missouri, Columbia, MO 65211 USA; 3 School of Agriculture and Biology, Shanghai Jiao Tong University, Shanghai, 200240 China; 4 Department of Computer Science, Informatics Institute, and Christopher S. Bond Life, Sciences Center, University of Missouri, Columbia, MO 65211 USA; 5 Department of Plant Biology, Carnegie Institution for Science, Stanford, CA 94305 USA; 6 Present address: Shandong University, Jinan, Shandong China; 7 Present address: Agronomy College of Shenyang Agricultural University, Shenyang, Liaoning China

**Keywords:** Soybean, GmBZL3, Brassinosteroid, ChIP-seq, Hormone crosstalk, SNP

## Abstract

**Background:**

Brassinosteroids (BRs) play a crucial role in plant vegetative growth and reproductive development. The transcription factors BZR1 and BES1/BZR2 are well characterized as downstream regulators of the BR signaling pathway in *Arabidopsis* and rice. Soybean contains four BZR1-like proteins (GmBZLs), and it was reported that *GmBZL2* plays a conserved role in BR signaling regulation. However, the roles of other GmBZLs have not been thoroughly studied, and the targets of GmBZLs in soybean remain unclear.

**Results:**

In this study, we first characterized GmBZL3 in soybean from gene expression patterns, conserved domains in coding sequences, and genomic replication times of four GmBZL orthologous. The results indicated that GmBZL3 might play conserved roles during soybean development. The overexpression of *GmBZL3*^*P219L*^ in the Arabidopsis BR-insensitive mutant *bri1–5* partially rescued the phenotypic defects including BR-insensitivity, which provides further evidence that GmBZL3 functions are conserved between soybean and the homologous Arabidopsis genes. In addition, the identification of the GmBZL3 target genes through ChIP-seq technology revealed that BR has broad roles in soybean and regulates multiple pathways, including other hormone signaling, disease-related, and immunity response pathways. Moreover, the BR-regulated GmBZL3 target genes were further identified, and the results demonstrate that GmBZL3 is a major transcription factor responsible for BR-regulated gene expression and soybean growth. A comparison of GmBZL3 and AtBZR1/BES1 targets demonstrated that GmBZL3 might play conserved as well as specific roles in the soybean BR signaling network. Finally, the identification of two natural soybean varieties of the *GmBZL3* mutantion by SNP analysis could facilitate the understanding of gene function during soybean development in the future.

**Conclusions:**

We illustrate here that GmBZL3 orchestrates a genome-wide transcriptional response that underlies BR-mediated soybean early vegetative growth, and our results support that BRs play crucial regulatory roles in soybean morphology and gene expression levels.

**Electronic supplementary material:**

The online version of this article (10.1186/s12870-019-1677-2) contains supplementary material, which is available to authorized users.

## Background

Brassinosteriods (BRs) play important and essential roles in a wide range of plant growth and development processes including cell elongation, cell division, plant architecture, photomorphogenesis, root development, photosynthesis, and senescence [1, 2]. BRs also play significant roles in controlling flowering time, male fertility, fruit ripening, seed development, seed filling and seed dormancy [3, 4]. In addition, BRs are essential for plant responses to various abiotic and biotic stresses, such as salt, drought, heat, cold, oxidative and heavy metal stresses, pathogen attacks and herbicide/pesticide tolerance [5, 6]. The mechanisms of BR biosynthesis, signaling and response at the molecular level have been well characterized through genetics, proteomics and genomics technologies in *Arabidopsis*. The expression of BR biosynthetic genes is regulated by feedback from BR signaling to maintain balanced cell expansion in normal plant development [7–9]. The transcription factors, BZR1 and BZR2/BES1 have been reported to directly bind and regulate the expression of downstream target genes in the BR synthesis feed-back loop [10–12]. When the BR level is low, BZR1 is phosphorylated by the upstream BR signaling regulator BIN2 and then retained in the cytoplasm by the 14–3-3 proteins or degraded by the proteasome, which allows the expression of BR biosynthetic genes and then increases the level of BR [13, 14]. When the BR level is high, dephosphorylated BZR1 translocates to the nucleus where it can bind to the target DNA regions and inhibits the expression of downstream BR-biosynthetic and BR-responsive genes [11, 15]. Several studies have linked BR signaling with numerous cellular processes by connecting the BR response genes and BZR1/BZR2 target genes [15, 16]. In conclusion, BZR1 and BZR2/BES1 are master transcription factors that mediate BR responsive gene expression, coordinating BR signaling, and BR biosynthesis and growth responses.

Soybean is a high protein and high oil crop that provides many products for humans and animals, including food. Several findings indicate that BRs play important roles during soybean growth and development. BRs dominate nodule formation in soybean roots and promote soybean epicotyl elongation [17–19]. The total nodulation, plant fresh weight, root length, shoot length, first internode length and lateral root numbers of soybean decreased in the 3 weeks after incubation with 0.1 μM to 10 μM of epibrassinolide [20]. Foliar application of BRs prior to drought stress could partially alleviate the detrimental effects of stress on the growth of soybean [21]. GmCPDs, genes that catalyze BR synthesis, are involved in the early stages of flowering regulation [22]. The pod size and plant height of the Arabidopsis mutant *bri1–5* can be complemented by overexpressing *GmBRI1*, which suggests that GmBRI1 could function as a BR receptor to mediate BR signaling [23, 24]. However, the gene regulatory network that links BR signals to various morphological and physiological responses remains largely unknown in soybean.

Recently, the function of *GmBZL2* (AtBZR1-like gene) was studied in soybean. Overexpressed GmBZL2^p216L^ Arabidopsis transgenic plants could partially rescue the defects of *bri1–5* mutant and increase the seed number per silique, which reveals the involvement of GmBZL2 in a conserved BR signaling regulation pathway in *Glycine max* [25]. It was reported that soybean varieties with larger pods usually have higher GmBZR1 (*GmBZL2*) expression levels in pods [26]. These results suggest that understanding the roles of GmBZL2 in soybean BR signaling pathways can offer valuable information for enhancing soybean productivity. However, soybean contains four *GmBZL* genes, and the functions of other *GmBZL* genes remain unclear. Moreover, genome-wide studies have revealed that BZR1 and BES1/BZR2 directly regulate thousands of target genes in *Arabidopsis*, revealing a regulatory network of plant growth regulation [15, 16]. A similar genome-wide study of GmBZL targets is required to elucidate the functions of BR in soybean.

In this study, GmBZL3 was first characterized in the soybean genome. The functions of *GmBZL3* in BR signaling were investigated by overexpressing GmBZL3 and GmBZL3^P219L^ in the Arabidopsis *bri1–5* mutant. Furthermore, our ChIP-seq analysis validated that GmBZL3 not only functions as a transcriptional regulator to mediate BR signal transduction but also functions as a hub to mediate BR crosstalk with other pathways. Finally, the natural variations in the *GmBZL3* coding sequence were explored using soybean whole genome resequencing data, providing a good resource for gene functional analysis in the future.

## Results

### Characterization of *GmBZL3* in the soybean genome

The four *GmBZLs* in *G. max* are divided into two subgroups internally based on phylogenetic tree analysis, which indicates that they may be from a specific duplication event during soybean evolution [25]. The soybean genome experienced segmental and tandem duplication events during evolution, causing gene family expansion, and the segmental duplication events in soybean occurred 59 and 13 million years ago (mya) [27]. Ka/Ks is the ratio between the number of nonsynonymous substitutions per nonsynonymous site (Ka) and the number of synonymous substitution per synonymous site (Ks). Here, the Ka/Ks ratios were calculated to evaluate the approximate duplication date of GmBZLs. We found that genome duplications of GmBZL1/2 and GmBZL3/4 clusters appear to have occurred at approximately 11 and 16 mya, respectively. Therefore, the duplication of GmBZL1/2 and GmBZL3/4 probably evolved through segmental duplication (Additional file 1). However, the duplicated GmBZL genes are under strong negative selection, as their Ka/Ks ratios were estimated to be < 1 (varied from 0.05 to 0.15).

Then, we analyzed the expression profiles of the *GmBZL* genes in different vegetative and reproductive tissues from published RNA-seq data sets [28]. As shown in the Additional file 2 A, *GmBZL3* transcripts accumulated at a high level in the pod, while *GmBZL2* was highly expressed in the flower and GmBZL1/4 were highly expressed in the shoot apical meristem. Several reports have confirmed that *AtBZR1* is engaged in various stress responses [5, 6, 29]. To gain more insight into the roles of *GmBZLs* under abiotic stress, gene expression patterns under various abiotic stress conditions (sodium, cold, dehydration and ABA) were further characterized using qRT-PCR (Additional file 2B-C). The results show that *GmBZL3* expression levels are increased under both salinity and dehydration stresses, suggesting a role under abiotic stresses through transcriptional regulation.

**Fig. 1:**
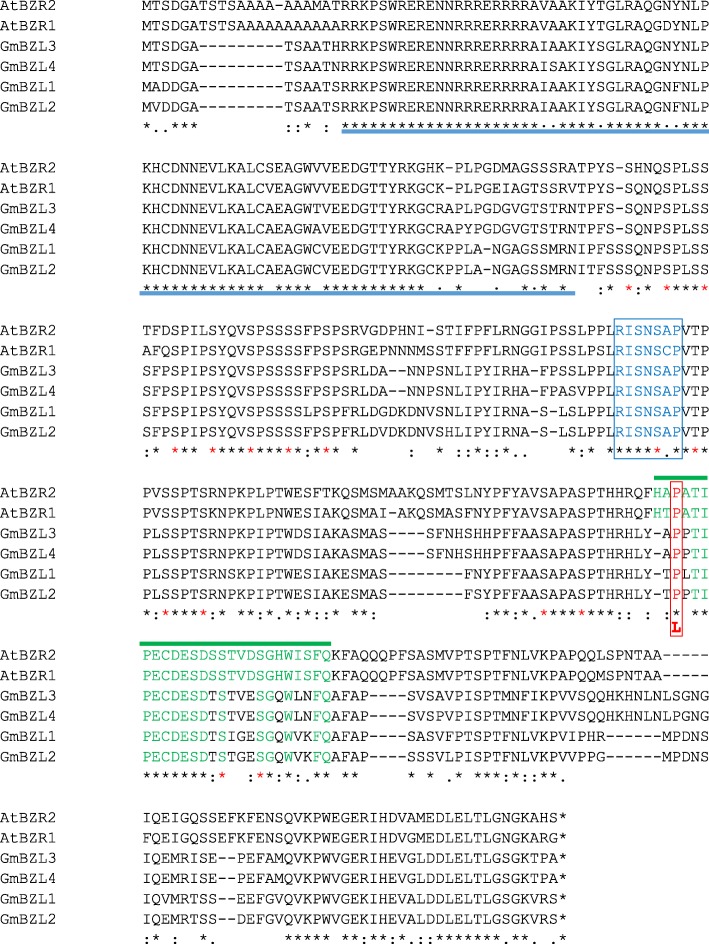
Multiple amino acid sequence alignment of the GmBZL in Soybean and Arabidopsis. The marked features are the N-terminal DNA binding domain (blue underline), putative 14–3-3 binding site (blue letters and box), putative sites of phosphorylation by GSK-3 kinase (red star), and the PEST domain (green letter and over line). The conserved amino acid (proline) between Arabidopsis and soybean in the PEST region is indicated by a red box, and was mutated to the amino acid leucine for the validation of conserved functional

To identify regions of similarity that may be a consequence of functional relationships, multiple amino acid sequence alignments were completed between *AtBZR1/BZR2* and *GmBZLs*. As shown in Fig. 1, GmBZL3 contains highly conserved sequences including N-terminal DNA-binding domains, putative 14–3-3 binding sites, and sixteen putative BIN2 phosphorylation sites. Furthermore, GmBZL3 also contains the PEST domain (region rich in proline, glutamate, serine, and threonine), which share high similarity with that of the Arabidopsis genes. The conserved amino acid proline (Fig. 1, highlighted with the red color and boxes), which affects protein localization and phosphorylation status, was found in the PEST region of GmBZL3. All of the above observations suggest that GmBZL3 plays a conserved role in the BR signaling pathway in soybean.

### Cross-species complementation test of *GmBZL3* in *Arabidopsis*

**Fig. 2:**
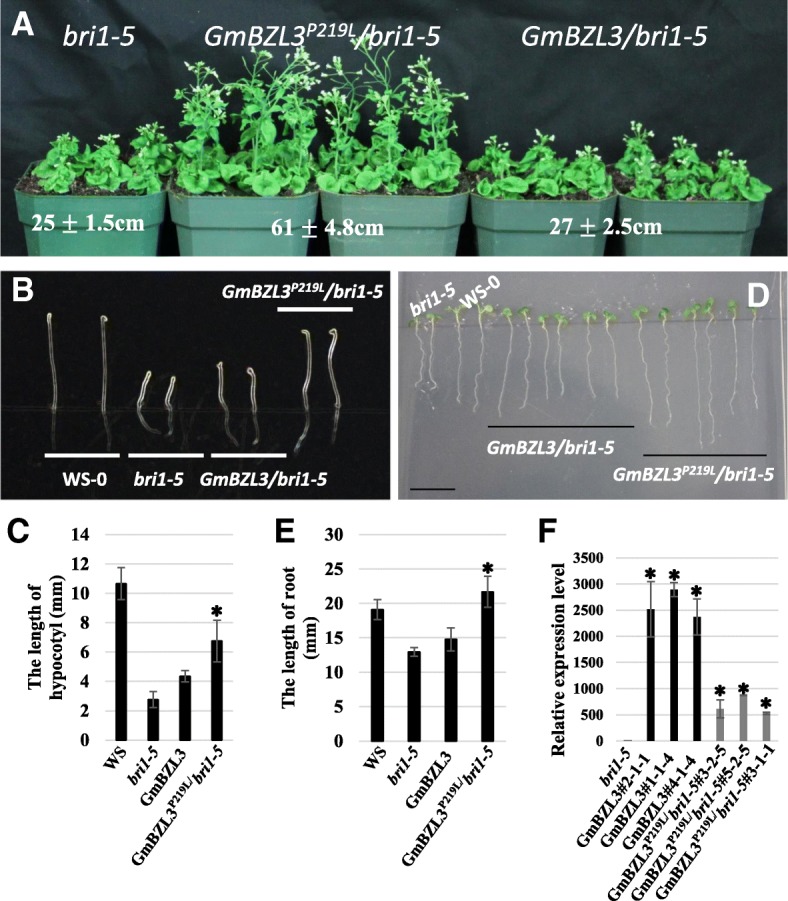
GmBZL3^P219L^ rescues the *bri1–5* insensitive phenotype in transgenic plants. GmBZL3/*bri1–5* indicates overexpressed GmBZL3 in the *bri1–5* mutant. GmBZL3#2–1-1, GmBZL3#1–1-4 and GmBZL3#4–1-4 are the three independent lines in which GmBZL3 is overexpressed in the *bri1–5* background. GmBZL3^P219^/*bri1–5* indicates overexpressed GmBZL3^P219^ in the *bri1–5* mutant. GmBZL3^P219^/*bri1–5*#3–2-5, GmBZL3^P219^/*bri1–5*#5–2-5 and GmBZL3^P219L^/*bri1–5*#3–1-1 are the three independent lines that overexpress GmBZL3^P219L^ in a *bri1–5* background. **a** The plant height of a one-month-old *bri1–5* mutant was significantly rescued by overexpressing GmBZL3^P219^. **b**-**c** Comparison of the length of the hypocotyl of 4-day seedlings (grown in the dark) in various plant lines. **d-e** Comparison of the length of the primary root of 7-day seedlings (grown under light) in various plant lines. f Transgene expression in various lines revealed by quantitative RT-PCR. Significant difference between the compared values are shown (*P* < 0.05). Error bars indicate S.D. (*n* ≥ 30)

A dominant *bzr1–1D* mutation increases BZR1 protein accumulation, suppresses BR-insensitive mutant (*bri1*) phenotypes, and enhances the feedback inhibition of BR biosynthesis in *Arabidopsis* [10]. The nuclear localization and phosphorylation ratio of GmBZL2 induced by BR and overexpressed GmBZL2^P216L^ (containing the conserved proline site mutation) in *Arabidopsis* Col-0 could produce the *bzr1–1D* phenotypes [25]. Cross-species complementation experiments were performed here to test whether the *GmBZL3* gene is a functionally conserved regulator in the BR signaling pathway. Transgenic Arabidopsis plants were generated to overexpress *GmBZL3* or *GmBZL3*^*P219L*^ (containing a proline-to-leucine mutation) in the *bri1–5* mutant. As indicated in Fig. 2, no distinct difference in morphological phenotype was observed between the *GmBZL3/bri1–5* transgenic plants and the *bri1–5* mutant (Fig. 2a). Furthermore, the *GmBZL3*/*bri1–5* plants showed a similar phenotype as the *bri1–5* mutant under either dark or light conditions during the seedling stage (Fig. 2b-e). However, the *GmBZL3*^*P219L*^/*bri1–5* transgenic plants showed a *bzr1–1D-*like phenotype with improved plant height at the mature stage under normal conditions (Fig. 2a). The *GmBZL3* ^P219L^*/bri1–5* seedlings had nearly double the hypocotyl length under dark conditions compared with that of *bri1–5* mutant seedlings (Fig. 2b and c). Under light conditions, the root growth of *GmBZL3* ^P219L^*/bri1–5* seedlings was longer than that of the *bri1–5* mutant (Fig. 2d and e). Interestingly, even higher RNA expression levels of *GmBZL3* in GmBZL3/*bri1–5* compared with GmBZL3^P219L^/*bri1–5* were found (Fig. 2f)*.* Taken together, these results demonstrate that GmBZL3 can regulate BR signaling when heterologously expressed in *Arabidopsis* and thus is functionally conserved.

**Fig. 3:**
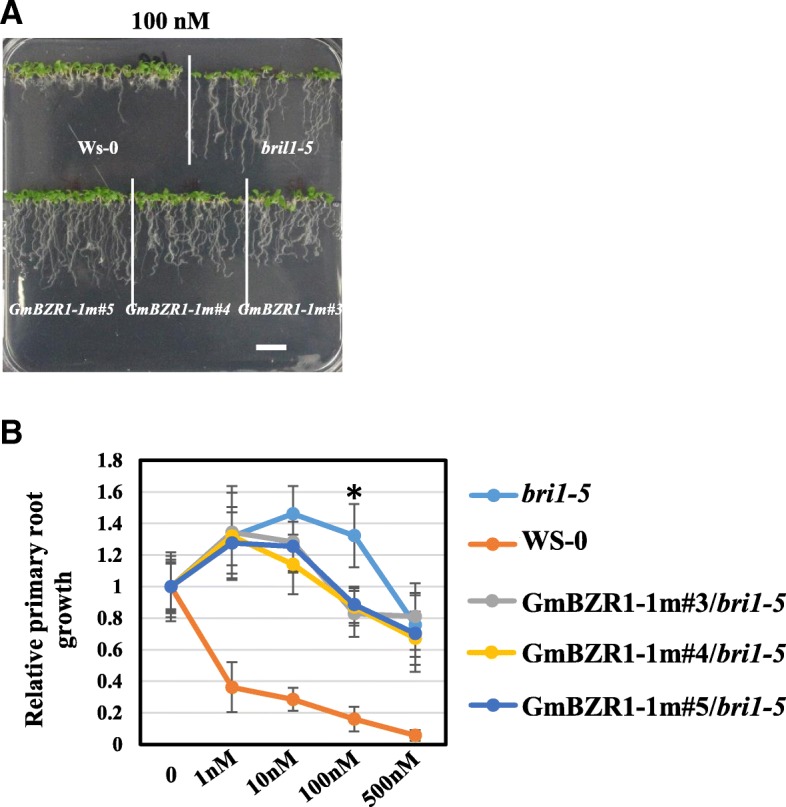
GmBZL3^P219L^/*bri1–5* transgenic plants show reduced insensitivity to BRs compared with *bri1–5*. Seedlings were grown under various concentration of epibrassinolide for 7 days in 16 h light/ 8 h dark condition. The root length was measured. Significant difference between the compared values are shown (*P* < 0.05). Error bars indicate S.D. (n ≥ 30)

Previous studies showed that AtBZR1 and GmBZL2 could partially rescue the BR-sensitivity phenotypes of *bri1–5* mutant in *Arabidopsis* [10, 25]. As shown in Fig. 3a, the root length of wild type was significantly inhibited by exogenous 100 nM BL. However, the root length of *bri1–5* was increased under lower concentrations of BL (1–100 nM). We also found that overexpressed *GmBZL3*^*P219L*^ partially rescued the *bri1–5* insensitivity phenotype at both 10 nM and 100 nM (Fig. 3b). The insensitivity phenotype was significantly rescued in the *GmBZL3*^*P219L*^*/bri1–5* seedlings compared with *bri1–5* under 100 nM BL treatments. This result illustrates that the conserved amino acid mutation (P219L) in the PEST motif of *GmBZL3* led to the altered BR response phenotypes.

### ChIP-seq results reveal that GmBZL3 regulates a broad range of biological processes and cellular activities

A ChIP-seq experiment was carried out to identify genes directly regulated by native GmBZL3 in soybean using an anti-GmBZL3 antibody. Since the pre-immunization serum was not used as a negative control, ChIP-qPCR analysis was conducted by using the immunoprecipitation of DNA before resequencing. Four genes (*DWF4*, *BR6ox2*, *CPD*, *BAS1*) that may act as GmBZL3 targets were selected. PROTEIN PHOSPHATASE 2A (PP2A) mediates the dephosphorylating and activation of BZR1 by protein interaction was used as negative control. As shown in Additional file 3, the amount of DWF4 fragment significantly enriched in the immunoprecipitation of DNA compared with that of PP2A. No significant difference was found in other three putative targets.

**Fig. 4:**
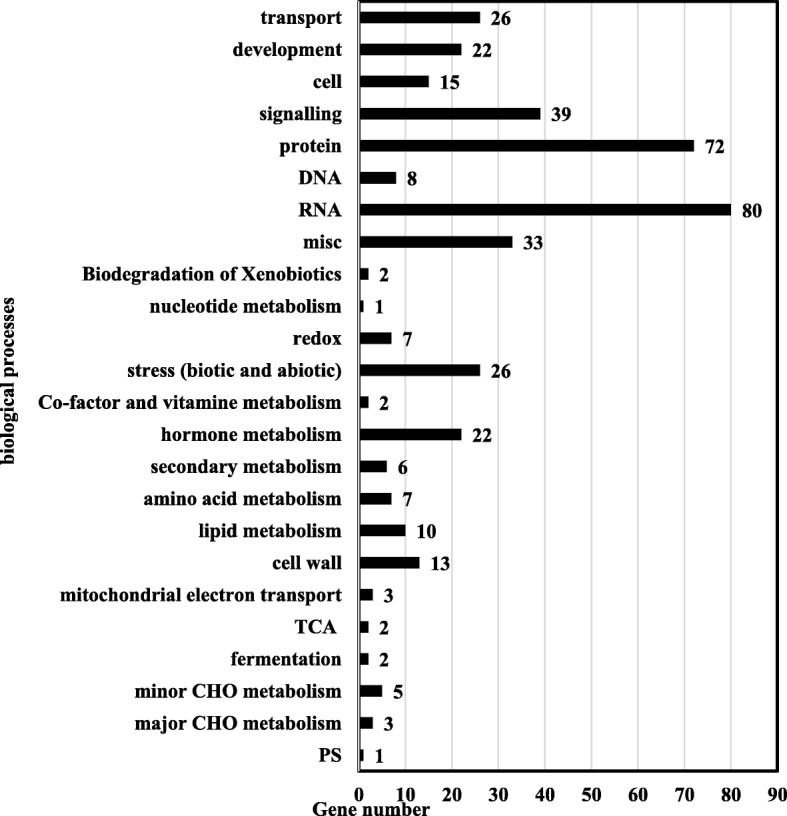
Functional categorization of GmBZL3-targeted genes based on biological processes. The identified high-ranking GmBZL3 targets are grouped based on MapMan “Bin” and GO ontology. RNA and protein processes related targets were significantly enriched. Gene numbers are displayed next to the terms. The abscissa indicates the number of targets in each category and the ordinate indicates the enriched GO categories in biological processes

Furthermore, 5185 binding peaks were identified in two independent biological repeats. These binding sites were linked to 2923 nearest neighbor genes, which were located on all 20-soybean chromosomes. Among these, 729 genes with high peaking scores were considered high-confidence GmBZL3-binding target genes (Additional file 4) and included in the subsequent studies. GO term analysis indicated that 407 genes were involved in different biological processes (Fig. 4), and 322 genes were not assigned to any known process. Among the enriched biological processes, RNA (regulation of transcription) and protein composed the largest subgroups. Genes related to transport (26), development (22) signaling (39), miscellaneous (33) and stress (26) represented the major categories, indicating that GmBZL3 plays multiple roles during soybean development. A small number of genes in the categories of secondary metabolism (6), amino acid metabolism (7), lipid metabolism (10), and carbohydrate metabolism (8) were also found. According to the molecular functional distribution, a total of seven categories were found to be highly and significantly enriched in genes involved in DNA binding, sequence-specific DNA binding transcription factor activity, protein binding, transport activity, etc., (Additional file 5, *p* < 0.05). These results indicate that GmBZL3 is a major transcription factor responsible for gene expression and soybean growth.

### GmBZL3 directly regulates genes that function in the synthesis and response of other hormones

Crosstalk between BR and other hormones has been observed at both the physiological level and the gene expression level in *Arabidopsis* [30–34]. However, functional interactions among hormones remain largely unknown in soybean. In this study, we found some genes related to auxin signaling, response and synthesis that were directly targeted by GmBZL3, including SAUR-like protein, GH3 and YUCCA3 (Table 1). Directly target genes were also been identified for ethylene metabolism and regulation of ethylene responsive pathways (Glyma.06G221800 and Glyma.18G164100). In addition, GmBZL3 targeted a number of gibberellin synthesis-degradation-related genes and GA-regulated family genes (Glyma.13G039600 and Glyma.13G069900). Several Jasmonate (JA) pathway-related genes, including *LOX2*, *AOC4* and *OPR2*, were identified in our ChIP-seq experiments. JAZ1 is a coreceptor and repressor of JA signaling in *Arabidopsis* [35]. Here, we found that GmBZL3 could bind the promoter region of two JAZ1 genes, which may indicate a negative relationship between BR and JA. These results demonstrated that the crosstalk between BR and other hormones existed in soybean and that GmBZL3 acts as an intermediate factor to mediate hormonal crosstalk.

**Table 1:** Representative target genes with high-ranking score (> 70) that potentially regulated by GmBZL3 transcription factor in soybean were identified through ChIP-seq method

Annotation	Identifier
Expansin	Glyma.06G246400 Glyma.02G109100 Glyma.06G195000 Glyma.14G203900
Auxin efflux carrier family protein	Glyma.13G038300
Auxin-responsive GH3 family protein	Glyma.02G125600
IAA-leucine-resistant (ILR1)-like 3	Glyma.04G226600
SAUR-like auxin-responsive protein family	Glyma.11G096800 Glyma.18G110300
YUCCA 3	Glyma.20G080000
Dormancy/auxin associated family protein	Glyma.20G237200
Jasmonate-zim-domain protein 1	Glyma.09G071600 Glyma.15G179600
Gibberellin 20-oxidase 3	Glyma.14G157400
Gibberellin 2-oxidase 4	Glyma.07G236100 Glyma.17G037300
Gibberellin 2-oxidase 8	Glyma.05G081600
Gibberellin-regulated family protein	Glyma.13G039600 Glyma.13G069900
BAK1-interacting receptor-like kinase 1	Glyma.03G095700 Glyma.08G062800
Brassinosteroid signalling positive regulator (BZR1) family protein	Glyma.06G034000
Disease resistance protein (CC-NBS-LRR class) family	Glyma.05G082200 Glyma.05G082500 Glyma.01G183400 Glyma.17G180300
Disease resistance protein (TIR-NBS-LRR class) family	Glyma.13G194900
Disease resistance-responsive (dirigent-like protein) family protein	Glyma.03G147900 Glyma.19G151100
LRR and NB-ARC domains-containing disease resistance protein	Glyma.03G039300
NB-ARC domain-containing disease resistance protein	Glyma.01G065800
SCARECROW-like 13	Glyma.07G266500
Senescence associated gene 20	Glyma.11G163100
Stachyose synthase	Glyma.19G217700
Fatty acid biosynthesis 1	Glyma.15G181500
Flowering promoting factor 1	Glyma.17G202100
Nitrate transporter 1.1	Glyma.05G056900 Glyma.01G042100 Glyma.02G022200
WRKY DNA-binding protein	Glyma.05G127600 Glyma.06G219800 Glyma.02G232600 Glyma.14G200200
WRKY family transcription factor	Glyma.13G117600 Glyma.19G254800
Dof-type zinc finger DNA-binding family protein	Glyma.15G071400 Glyma.15G082400 Glyma.19G199200
NAC (No Apical Meristem) domain transcriptional regulator superfamily protein	Glyma.05G195000 Glyma.06G248900 Glyma.12G149100 Glyma.12G226500
NAC transcription factor-like 9	Glyma.06G138100
PIF1 helicase	Glyma.10G079800 Glyma.13G024400 Glyma.14G128000
DnaJ/Hsp40 cysteine-rich domain superfamily protein	Glyma.14G104200
Heat shock cognate protein 70–1	Glyma.03G171100
Heat shock protein DnaJ with tetratricopeptide repeat	Glyma.19G215400
Heat shock transcription factor B2A	Glyma.16G196200

### GmBZL3 mediates crosstalk between BR and other development pathways

In *Arabidopsis*, the hormonal interactions between BR and abscisic acid, ethylene or salicylic acid participate in stress-related development [29]. However, the molecular mechanism of the involment of BR in stress tolerance remains poorly understood in soybean. It has been reported that BZR1 mediates the antagonism between immunity signaling and BRs by inducing the expression of several WRKY transcription factors that negatively control early immune responses [36]. Here, we found that several disease resistances-related genes and WRKY transcription factors were targeted by GmBZL3. BR controls plant growth by acting on both expansion and division in the leaf [37]. The AtBZR1 protein accumulates in the growing region of Arabidopsis hypocotyl under dark conditions, indicating that BZR1 plays a role in cell expansion [10]. Studies of the Arabidopsis *bzr1–1D* mutant suggested that BZR1 is involved in growth promotion [10]. Here, we found that GmBZL3 directly regulated the expression of four expansin genes (Table 1). Several targets involved in drought and heat stress responses, such as heat shock protein, heat shock transcription factor, and ERD15 (early responsive to dehydration 15), were found. Moreover, senescence, fatty acid biosynthesis and stachyose synthase related genes were regulated by GmBZL3. These results not only confirmed that a similar BR regulatory mechanism existed in *Arabidopsis* and soybean but also suggested that the transcription factor GmBZL3 directly linked BR signal transduction with soybean development.

### The expressions of GmBZL3 targets were tightly regulated under different BR levels in soybean

Propiconazole (Pcz), a BR biosynthesis inhibitor, inhibits BR metabolism and induces BR deficiencies in *Arabidopsis*, maize and soybean seedlings [38], (Song et al., unpublished). The expression levels of BR response genes are precisely regulated by BR concentration [11, 12, 16]. Genome-wide gene expression analysis was conducted by using stage V1 (first-node: fully developed leaves at unifoliolate node) soybean seedlings exposed to higher concentrations of BR synthesis inhibitor with/without epi-brassinolide. Briefly, V1 stage soybean plants were irrigated with water containing high concentration BR synthesis inhibitor (5 μM propiconazole for 10 days, Banner Maxx-60,207-90-1, Syngenta, Greensboro, NC), or a combined treatment of high concentration inhibitor and low concentration brassinolide (BL) (5 μM Pcz with 10 nM BL for 10 days, Pcz-BL). High concentration BL was applied for 1 h or 8 h after 10 days treatment with high concentration inhibitor(5 μM Pcz for 10 days then with 1 μM BL 1 h, Pcz-BL-1 h; 5 μM Pcz for 10 days then with 1 μM BL for 8 h, Pcz-BL-8 h) (Song et al., unpublished). To characterize the genome-scale adjustment of GmBZL3 targets during soybean development, the overlap between GmBZL3 targets and BR-regulated genes was examined.

**Fig. 5:**
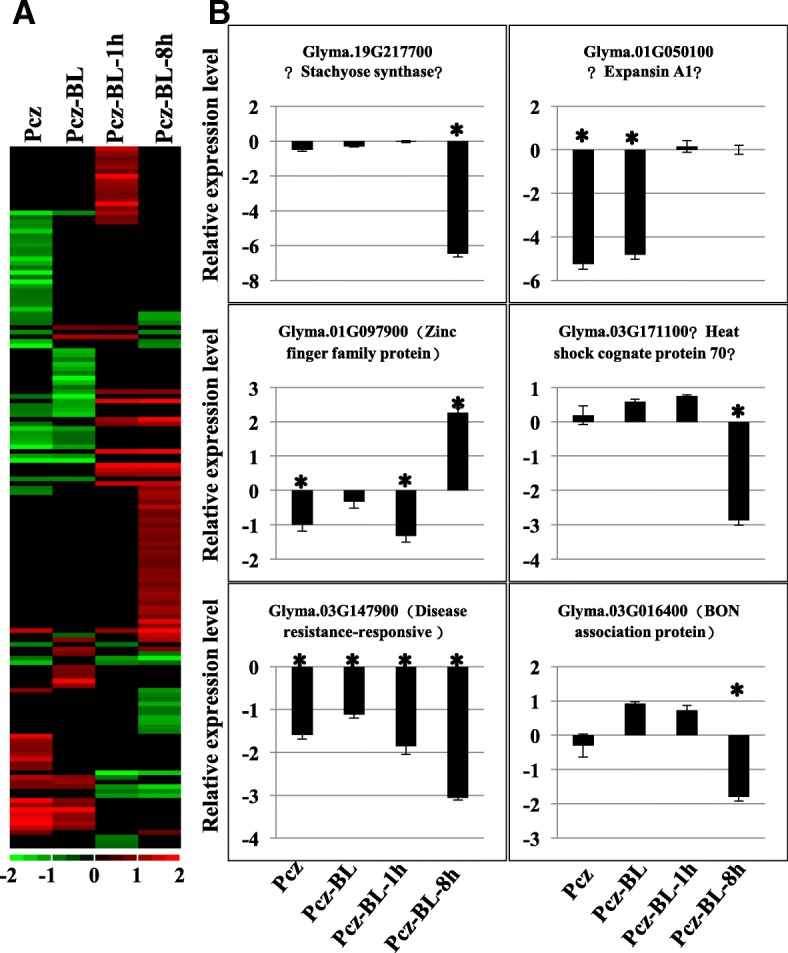
Expression patterns of GmBZL3 target genes in response to a BR inhibitor in combination with or without epibrassinolide. **a** Heatmap representation of expression patterns of different GmBZL3 targets in soybean Williams 82 under following conditions (Pcz: 5 μM Pcz for 10 days. Pcz-BL: 5 μM Pcz with 10 nM BL for 10 days. Pcz-BL-1 h: 5 μM Pcz for 10 days then with 1 μM BL for 1 h. Pcz-BL-8 h: 5 μM Pcz for 10 days then with 1 μM BL for 8 h). The expression data values were median-centered and normalized for each gene before transforming to the color scale (log2-transformed ratios). The color bar at the bottom shows the range of expression values from highest expression level (red) to lowest expression level (green). 0 is the median expression level (Black). **b** qRT-PCR analysis of six GmBZL3 target genes was performed using total RNA isolated from Wm82 seedlings under control. Pcz. Pcz-BL. Pcz-BL-1 h and Pcz-BL-8 h treatments. Relative gene expression levels (fold change, log2) are shown following normalization with actin (Glyma.18G290800) transcript values. Error bars represent the standard error of the mean. The y-axis represents the relative gene expression level in different samples. Three independent experiments were performed. A representative result is shown. The star (*) indicates statistically significant differences among the means (*p* < 0.05)

We found that the transcript abundance of 154 targets was significantly altered under at least one treatment condition (Fig. 5a). In addition, the transcript levels of some GmBZL3 targets were induced, while others were repressed under the same concentration of BR. Moreover, a number of genes were significantly differentially regulated under a specific treatment, but there was no obvious change in other treatments. Six target genes of GmBZL3 were further selected to verify the expression pattern at different BR levels using qRT-PCR. As shown in Fig. 5b, a stachyose synthase gene (Glyma.19G21770) and a heat shock protein (Glyma.03G171100) were significantly reduced by 5 μM Pcz followed by 1 μM BL for 8 h. An expansin gene (Glyma.01G050100) was reduced under 5 μM Pcz with or without I nM BL combination treatment. However, the expression of this expansin gene could be recovered by applying 1 μM BL for 1 or 8 h. These results indicated that the expression patterns of GmBZL3 targets were specifically controlled by different BR levels in soybean and that GmBZL3 plays an important regulatory function during the soybean response to different BR levels.

### Comparison of GmBZL3 and AtBZR1/BES1 targets

**Fig. 6:**
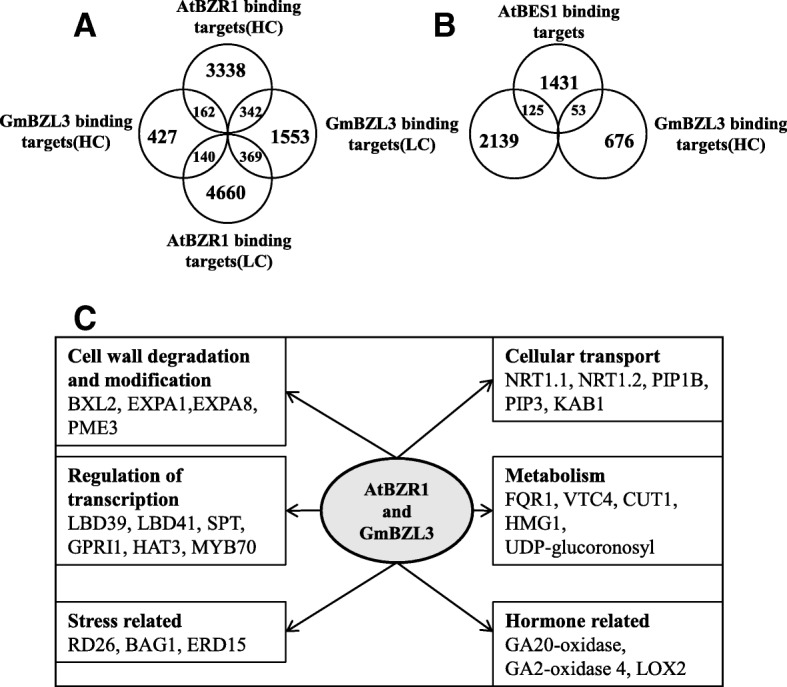
Comparison of GmBZL3 and AtBZR1/BES1 targets. **a-b** Venn diagram showing the overlap of GmBZL3 target genes with BZR1 or BES1 targets. HC: high-confidence. LC: low-confidence. **c** Representative overlaps of GmBZL3 target genes with BZR1 targets in various cellular, response and metabolic pathways. NRT1.1 (Nitrate transporter 1.1). NRT1.2 (Nitrate transporter 1.2). PIP1B (Plasma membrane intrinsic protein 1;2). PIP3 (Plasma membrane intrinsic protein 3). KAB1 (Potassium channel beta subunit). FQR1 (Flavodoxin-like quinone reductase 1). VTC4 (Inositol monophosphatase family protein). CUT1 (Cuticular 1). HMG1 (3-hydroxy-3-methylglutaryl coa reductase). LOX2 (Lipoxygenase 2). BXL2 (Beta-xylosidase 2). EXPA1 (Expansin A1). EXPA8 (Expansin A8). PME3 (Pectin methylesterase 3). LBD39 (lateral organ boundaries domain protein 39). LBD41 (lateral organ boundaries domain protein 41). SPT (SPATULA). GPRI1 (GOLDEN2-like 1). HAT3 (Homeobox-leucine zipper protein 3). AtMYB70 (Myb domain protein 70). RD26 (Responsive to dessication 26). BAG1 (BCL-2-associated athanogene 1). ERD15 (Early responsive to dehydration 15)

ChIP-chip studies have determined that BZR1 and BZR2/BES1 directly regulate over thousands of genes, revealing the brassinosteroid transcription network in *Arabidopsis* [15, 16]. To reveal the evolutionary variation of the BR gene network between *Arabidopsis* and soybean, we first compared 3410 BZR1 high-confidence targets with 729 GmBZL3 high-confidence target homologs. Our results showed that 162 out of 729 (22.2%) GmBZL3 targets were identical to BZR1 targets (Fig. 6a). Furthermore, 140 out of 729 (19.2%) GmBZL3 target genes were overlapped with BZR1 low-confidence targets. Therefore, a total of 302 (41.4%) GmBZL3 high-confidence target homologs were matched with BZR1 targets. However, only 53 (7.27%) overlapping genes were found between GmBZL3 target homologs and BES1 targets (Fig. 6b). Together, these analyses demonstrated that GmBZL3 might play conserved as well as specific roles in the soybean BR signaling network similar to the role of BZR1 in *Arabidopsis*. Furthermore, the small number of overlapping targets between GmBZL3 and BES1 may imply that there are different functions between soybean and *Arabidopsis*.

The Analyses of these overlapping genes between the GmBZL3 target homologs and the BZR1 targets showed a wide range of cellular transport, cell wall-related enzymes, transcription factors, metabolism, stress, and hormone-related genes (Fig. 6c). For example, EXPA1 and EXPA8, which likely mediate the BR responses of cell elongation and differentiation, were targeted by both GmBZL3 and BZR1. The gibberellin synthesis-degradation-related genes, GA20-oxidase and GA2-oxidase were regulated by both GmBZL3 and BZR1. These results indicated that several developmental processes are conserved in the BR response networks of soybean and *Arabidopsis* and revealed the nodes of crosstalk between BR and other regulatory pathways.

### Exploring natural variations in the *GmBZL3* coding sequence using soybean whole genome resequencing data

To understand the genetic variations of *GmBZL3* in different soybean varieties, single nucleotide polymorphisms (SNPs) were identified in 106 soybean genotypes by using whole genome resequencing data [39]. Only two synonymous SNPs were observed in the coding region of *GmBZL3* compared with the reference genome sequence (Williams 82) (Additional file 6). However, one nucleic acid deletion was found in the soybean lines PI594599 and PI603154. This nucleic acid gap causes an amino acid frame shift and introduces a premature stop codon in these two soybean genotype lines. Specifically, the conserved PEST sequence in *GmBZL3* can be completely changed due to this deletion. These identified soybean lines can be used as mutant sources for further functional characterization of the GmBZL3 gene in the soybean BR signaling pathway.

## Discussion

We illustrate here that GmBZL3 orchestrates a genome-wide transcriptional response that underlies BR-mediated soybean early vegetative growth, and our results support that BRs play crucial regulatory roles in many processes ranging from morphology to gene expression levels. A cross-species complementation test in *Arabidopsis* demonstrated that GmBZL3 plays conserved and broad roles in regulating BR signaling.

### Gene sequence and structure characteristics of *GmBZL3*

*AtBZR1* and *AtBES1/BZR2* in *Arabidopsis* have a high similarity of 88% identity in overall amino acid sequence and 97% identity in the N-terminal DNA binding domain [10]. In addition, *AtBZR1* and *AtBZR2/BES1* bind to similar DNA sequences, such as the E-box (CANNTG) and BRRE (CGTGT/CG) motifs [11, 12, 16]. These findings of high sequence similarity indicate that the *BZR1* gene family may play conserved biological functions. In soybean, four *GmBZR1* orthologues show similar sequence domains to *AtBZR1* and *AtBES1/BZR2*. It was reported that the PEST sequence serves as a proteolytic signal regionthat targets proteins for rapid degradation [40]. A PEST sequence located in the BZR protein sequence was identified in *Arabidopsis*, rice and soybean [10, 25, 41]. A proline-to-leucine mutation in the PEST domain leads to the accumulation of BZR1 and altered BR responsive phenotypes in *bzr1–1D* and *bes1-D* mutants [41]. The PEST sequence mutation in bzr1–1D increases its interaction with PP2A phosphatase and enhances dephosphorylation of the BZR1 protein [42], which reduces its binding affinity to the 14–3-3 protein as well as increases its nuclear accumulation and DNA binding [13]. The bzr1–1D allele reverses the dwarf phenotype when transformed into the *bri1–5* mutant, whereas the overexpression of the wild type *BZR1* in either WT or *bri1–5* mutant plants did not cause obvious phenotypic changes due to the tight control of BZR1 activity by phosphorylation [10]. The effect of a conserved mutation located in the PEST motif of GmBZL3 was confirmed through functional validation in transgenic *Arabidopsis* plants (Fig. 3). The C-terminal domain is required for interactions between BZR2/BES1 and BIN2 or BIM1 in vivo [12, 43]. In this study, 16 putative BIN2 phosphorylation sites were identified in GmBZL3. Shortened hypocotyls, roots, and stems are the most notable morphological characteristics of the Arabidopsis BR receptor mutants. The overexpression of *GmBZL3* ^P219L^ could rescue the stem, hypocotyl, and root lengths, but could not rescue the BR insensitivity, as expected. These results demonstrate that GmBZL3 is a functional transcription factor involved in the BR signal transduction cascade.

### GmBZL3 targets response to BR signaling

A large numbers of overlapping target genes regulated by AtBZR1 and AtBZR2/BES1 were identified by combining the chromatin-immunoprecipitation microarray (ChIP-chip) and RNA-seq/microarray results [15, 16]. Genome-wide studies have further revealed that the BES1/BZR1 family regulates a transcription network involving thousands of genes. Approximately one-quarter of BR-responsive genes are directly regulated by BZR1/BZR2 (approximately 1000 genes) [15, 16]. In our study, more than two thousand target genes bind by GmBZL3 were identified through ChIP-seq experiments, which indicated that GmBZL3 is a major regulator of the BR pathway. In addition, many GmBZL3 targets showed increased or decreased of expression levels in response to different BR levels based on four sets of RNA-seq data. Therefore, it is conceivable that GmBZL3 has a dual regulatory function under different BR levels.

### Comparison of GmBZL3 and BZR1 targets reveals interesting features of gene regulation

It has been reported that BR is involved in drought stress in a range of plant species [44–46]. BR signaling transduction-related receptors and regulators play important roles under drought conditions [47–49]. Recently, it was found that a NAC transcription factor, RD26 (Responsive to desiccation 26), mediates crosstalk between drought-related and BR signaling pathways by coordinating the transcriptional activities of RD26 and BES1 [50]. Two soybean genes (Glyma.06G248900 and Glyma.12G149100) encoding RD26 were targeted by GmBZL3 in our results and were significantly induced under Pcz and Pcz-BL treatment (data not shown). Previous studies indicated that ERD15 is a negative regulator of the ABA-response gene in *Arabidopsis* [51]. Here, we also found that ERD15 was targeted by GmBZL3 and BZR1 in soybean and *Arabidopsis*, respectively. Moreover, GmBZL3 and BZR1 targeted two water channel proteins (PIP1B and PIP3) in soybean and *Arabidopsis*, respectively. Therefore, our data not only support the conclusion that GmBZL3 plays a role in soybean drought response, but also provide candidate genes for elucidating the molecular mechanism in the future.

The de-etiolation phenotype of BR mutants indicates that BR plays an essential role in photomorphogenesis and many of the genes regulated by BZR1 overlap with the targets of HY5 and PIL5 (light-signaling pathway key transcription factor) [16, 52]. However, few light-related genes were found in GmBZL3 targets. This result demonstrates that GmBZL3 may play indirect and specific roles in the light signaling pathway.

### Soybean whole genome sequencing reveals good mutation resources for *GmBZL3*

It is difficult to validate gene functions by transgenic methods since soybean transformation efficiency is low [53]. Therefore, soybean genes are often transformed into other plants, such as *Arabidopsis* or tobacco, for functional evaluation. To date, hairy root transformation has offered an effective alternative to obtain a high number of soybean transformants in a relatively short time for gene function validation. Although this system has made significant contributions to soybean root studies, it is questionable in the case of characterization of non-root traits [54–56]. Luckily, there are several mutations in nature, such as a null soybean mutant or recessive alleles for target genes, which can be used as valuable genetic sources. The first complete reference genome for soybean, Williams 82, was released in 2010 [27]. Hyten et al. [57] developed an initial version of SNP detection assays to identify a large number of SNPs and constructed a genetic map with higher resolution. A large amount of genome sequencing data has been generated with recently published GWAS studies [39, 58, 59]. These genomic resources will provide a range of opportunities for soybean improvement through understanding gene function by map-based cloning and reverse genetic approaches.

In our study, two mutant lines of *GmBZL3* were identified by analyzing whole genome resequence data. These lines will be excellent materials for further gene functional characterization. Moreover, the marker-assisted backcrossing strategy can be used to develop *GmBZL3* mutants suitable for gene function validation. It has been reported that the BC4 F1 seeds have 99.0% recurrent parent genome after four rounds of backcrossing [60]. In the future, the analysis of genome sequencing data of all BR signaling-related factors and the examination of specific correlations between SNPs and traits in different germplasm lines can help reveal additional mechanistic details of BR and the interactions between interdependent pathways.

## Conclusion

BRs play crucial regulatory roles during soybean vegetative development. Our study demonstrated that GmBZL3 plays a conserved role in the soybean BR response pathway. Our results provide a genomic map of GmBZL3 actions in soybean revealing a regulatory network that which integrates hormonal and multiple regulatory pathways for plant growth and reveals numerous molecular links between the BR signaling pathway and downstream components involved in developmental and physiological processes. In addition, the natural variation in germplasm lines identified here would provide good mutations resources for gene functional analysis in the future.

## Methods

### Expression profiling analysis using RNA-seq datasets

The RNA-seq data generated by Libault et al. [28] from nine different soybean tissues (Williams 82 genotype) including flowers, leaves, nodules, pods, roots, root hairs, seeds, shoot apical meristems and stems, was used to analyze the expression patterns of four orthologous GmBZLs. Hierarchical clustering of expression data was performed using dCHIP software [61]. The RPKM value shown presents the normalized total reads obtained in each tissue library. For salt or ABA treatments, the soybean seedling (V1 stage) roots were immersed in solutions containing 200 mM NaCl or 100 μM ABA under room temperature with water as a control. For cold treatment, seedlings were kept at 4 °C with 16 h light/8 h dark. For dehydration treatment, seedlings were transferred onto filter paper, and dried at room temperature with 60% humidity. First trifoliate leaves from three plants after 8 h treatment were harvested for RNA isolation. Tissue samples were collected from three biological replicates, and three independent experiments were repeated. Four sets of RNA-seq data with BR synthesis inhibitor treatments were used to analyze the expression patterns of GmBZL3 targets (Song et al., unpublished). Briefly, soybean seedlings at the V1 stage were treated with a high concentration BR synthesis inhibitor (5 μM propiconazole for 10 days, indicated as **Pcz**) or were simultaneously treated with a high concentration inhibitor and low concentration brassinolide (BL) (5 μM Pcz with 10 nM BL for 10 days, indicated as **Pcz-BL**). Moreover, a high concentration of BL was applied in a short time after 10 days of treatment with a high concentration inhibitor (5 μM Pcz for 10 days then with 1 μM BL 1 h, indicated as **Pcz-BL-1 h**; 5 μM Pcz for 10 days then with 1 μM BL for 8 h, indicated as **Pcz-BL-8 h**).

### Protein sequence alignments and gene duplications of GmBZLs

Multiple sequence alignments were constructed using ClustalW2 (https://www.ebi.ac.uk/Tools/msa/clustalo/). The Ks and Ka values were extracted from the Plant Genome Duplication Database (PGDD) [62], and the values were used to calculate the approximate dates of duplication events. The date of duplication events was subsequently estimated according to the eq. T = Ks/2λ, in which the mean synonymous substitution rate (λ) for soybean is 6.1 × 10^− 9^ [63].

### Gene cloning, site-directed mutagenesis and genetic transformation

The full-length open reading frame (ORF) of the *GmBZL3* gene was amplified from Williams 82 with the following primer pairs: Glyma06g03700F: CACCATGACTTCGGACGGAGCAAC and Glyma06g03700R: AGCAGGCGTCTTCCCACTTCCAAGT. Then the PCR products were cloned into the pENTR™ D/TOPO vector (Thermo Fisher Scientific, Waltham, MA, USA). The positive clone was fully sequenced with M13 sequencing primers. An LR gateway reaction was performed with the binary vector pEarleyGate 101 [64]. The Phusion Site-Directed Mutagenesis Kit (Thermo Scientific) was used to create the *BZL3* ^*P219L*^ mutant gene containing the Pro-219-Leu [42] mutation site with the following primers: 14–3F: GAGGATATCAAACGCTGCCCCTGTTACCC; 14–3R: AAAGGAGGGAGAGACGAGGGAAACGCAT. The *GmBZL3*-pEarleyGate 101 and *GmBZL3*^*P219L*^-pEarleyGate 101 binary vectors were used for Arabidopsis transformation.

### Plant material, growth condition, and treatments

We received the Arabidopsis lines *bri1–5* and Ws-0 from Zhiyong Wang (Carnegie Institution for Science, Stanford, Wang et al., 2002 [10]). *GmBZL3* and *GmBZL3*^*P219L*^ were overexpressed in the Arabidopsis BR-insensitive mutant *bri1–5*. The *Agrobacterium*-mediated floral dip transformation method was employed to generate transgenic plants [65]. T3 homozygous transgenic lines were screened and used for the following phenotype analysis. Ws-0, *bri1–5*, overexpressed GmBZL3/*bril1–5* and overexpressed *GmBZL3*^P219L^/*bril1–5* lines were planted and grown on ½ solid MS media with 2% sucrose. For seedlings grown in the dark, the seeds were treated in cold for 2 days and then grown at 22 °C for 4 days in the dark. For light grown seedlings, cold treated seeds were grown at 22 °C for 6 days under a 14/10 h light/dark photoperiod and 100 μmol/m^2^/s light intensity. For the analysis of *GmBZL3* and *GmBZL3*^P219L^ gene expression levels in Arabidopsis transgenic plants, seedlings grown for 7 days in the light were used for RNA isolation. For BR response evaluation, cold treated seeds were grown at 22 °C for 7 days under a 14/10 h light/dark photoperiod on ½ solid MS media with varied BL concentrations (0,1 nM, 10 nM, 100 nM, and 500 nM).

### GmBZL3 antibody and genome-wide chromatin immunoprecipitation

The peptide (FAPSVSAVPISPT) from 244 to 256 of the deduced amino acids sequence of GmBZL3 was used for antibody production in rabbits. Antibodies were produced by Pierce Company (Thermo Scientific, Huntsville, AL). ChIP assays were performed according to the published protocol [66]. Briefly, 8 g of soybean leaves (V3 stage) were grounded and cross-linked with 1% (*v*/v) formaldehyde under a vacuum for 5 min, and then cross-linking was quenched by adding glycine to a final concentration of 0.25 M. The sample was kept under vacuum was continued for 5 more minutes. The cross-linked tissue was ground into powder in liquid nitrogen, and 5 g of tissue powder was used to isolate the chromatin complexes. Subsequently, polyclonal GmBZL3 antibodies were sonicated and immunoprecipitated. DNA was extracted by using a Fermentas PCR Purification kit. The precipitated DNA was resuspended in 50 μl TE and stored at − 20 °C for ChIP-seq analysis.

### ChIP sequencing and analysis

Two independent biological replicates were used for ChIP analysis. The DNA extracted from soybean leaves without ChIP treatment was used as a negative control. ChIP sequencing library construction and sequencing were conducted by University of Missouri DNA Core Facility using a Hi-seq 2000 (Illumina, San Diego, CA). The data analysis was performed as follows: the paired end reads were aligned to the soybean genomes using Bowtie [67]. The mapping results were saved as BAM files [68]. The significantly enriched ChIP regions were identified by the peak-calling program MACS, and the results were saved as the coordinates of the identified peaks on the genome [69]. The gene-calling tool (bedtools) was then used to characterize the genes around the peak regions [70].

### Quantitative real-time PCR assays

Total RNA was isolated from either soybean leaf or Arabidopsis seedlings with an RNeasy Plant mini kit (Qiagen, Cat#:74904). On-column DNase digestion with the RNase-Free DNase set was performed to remove DNA contamination (Qiagen, Valencia, CA, Cat#:79254). A High Capacity cDNA Reverse Transcription Kit (Thermo, USA, Cat#4368814) was used for cDNA synthesis. The qRT-PCR and ChIP-qPCR assays were carried out using SYBR Green master mix (Thermo, USA, Cat# K0223). The comparative Ct method was used to quantify the relative expression of specific genes [71]. The cyclin gene (Glyma10g263500) was selected as an internal control to normalize gene expression. All primers were designed using the Primer3 web interface (http://bioinfo.ut.ee/primer3-0.4.0/; [72]). Three biological replicates and repeated once as a technical replicate were performed in each reaction. The overexpression of the transgene was confirmed by using the primers GmBZL3-RTF(GGTCGTTTAATTGGAGGAGAAT) and (GmBZL3-RTR GATGAGGCCTATCATTTCCTG), which spanned the linking region between YFP and the transgene to exclude the possibility of Arabidopsis endogenous gene amplification. pEarly101-YFP-F (GTAAACGGCCACAAGTTCAG) and pEarly101-YFP-R(ACTTCAGGGTCAGCTTGC) were used for the gene expression level analysis in the transgenic Arabidopsis plants. The cyclin gene was chosen as an internal control by using primers as: Gm10g263500-RTF (ACCACAATGACCAACTAGAGC) and Gm10g263500-RTR (CTTCCTCTTCCCACTTTCCTTC). The PP2A gene was chosen as ChIP-qPCR internal control. Primers of ChIP-qPCR assay are listed in Additional file 7.

## Additional files

Additional file 1: Estimates of the dates for the segmental duplication events of the BZL gene pairs in soybean. (XLSX 9 kb)

Additional file 2: Expression patterns of *GmBZLs* in different tissues and under abiotic stress conditions. (A) Expression profiles of GmBZL genes in 9 tissues. The relative expression data of 9 tissues determined by RNA-seq were obtained from Libault et al.. 2010 and used to construct the expression patterns of soybean genes. Color in the heatmaps represents RPKM values of the GmBZL genes. (B-C) Transcription levels of GmBZL genes in response to abiotic stress. The soybean V1 stage seedlings were treated with 200 mM NaCl or 100 μM ABA for 8 h. For cold treatment, seedlings were kept at 4 °C with light. For dehydration treatment, seedlings were transferred onto filter paper and dried at room temperature with 60% humidity. Relative gene expression levels are shown following normalization with actin transcript values. Error bars represent the standard error of the mean. The star (*) indicates statistically significant differences among the means (*p* < 0.05). (PPTX 88 kb)

Additional file 3: DWF4 was significantly enriched in both IP biological repeat samples compared with PP2A, but no significant different of amount between DWF4 and PP2A in negative ChIP experiment. (PPTX 67 kb)

Additional file 4: ChIP data reveal genes potentially regulated by the GmBZL3 transcription factor in soybean. (XLSX 38 kb)

Additional file 5: Overrepresented GO categories in GmBZL3 targets. Functional classification of GmBZL3 targets indicated that seven sets of GO (molecular function) terms were significantly enriched (*p* values < 0.01). (PPTX 40 kb)

Additional file 6: Identification of nonsynonymous SNPs and deletions in the GmBZL3 gene from 106 soybean resequencing datasets (Valliyodan et al. 2016). Highlighted text showing SNPs and nonsynonymous mutations. (XLSX 13 kb)

Additional file 7: The list of primers used in qRT-PCR and ChIP-qPCR. (XLSX 10 kb)

## Abbreviations

AtMYB70: Myb domain protein 70
BAG1: BCL-2-associated athanogene 1
BC4: Back cross
bHLH: Basic helix-loop-helix
BL: Brassinolide
*bri1*: BR-insensitive mutant
BRs: Brassinosteroids
BXL2: Beta-xylosidase 2
ChIP-chip: Chromatin-immunoprecipitation microarray
ChIP-seq: Chromatin Immunoprecipitation Sequencing
CUT1: Cuticular 1
ERD15: Early responsive to dehydration 15
EXPA1: Expansin A1
EXPA8: Expansin A8
FQR1: Flavodoxin-like quinone reductase 1
GH3: Gretchen Hagen 3
GmBZLs: BZR1-like proteins
GPRI1: GOLDEN2-like 1
GWAS: Genome-wide association study
HAT3: Homeobox-leucine zipper protein 3
HMG1: 3-hydroxy-3-methylglutaryl coa reductase
JA: Jasmonate
JAZ1: Jasmonate ZIM-domain protein 1
KAB1: Potassium channel beta subunit
LBD39: Lateral organ boundaries domain protein 39
LBD41: Lateral organ boundaries domain protein 41
LOX2: Lipoxygenase 2
Mya: Million years ago
NRT1.1: Nitrate transporter 1.1
NRT1.2: Nitrate transporter 1.2
OPR2: 12-oxophytodienoic acid reductases 2
ORF: Open reading frame
Pcz: Propiconazole
PGDD: Plant Genome Duplication Database
PIP1B: Plasma membrane intrinsic protein 1;2
PIP3: Plasma membrane intrinsic protein 3
PME3: Pectin methylesterase 3
qRT-PCR: Quantitative real-time PCR
RD26: Responsive to desiccation 26
RD26: Responsive to dessication 26
RPKM: Reads per kilo base
SAURs: Small auxin-up RNAs
SNPs: Single nucleotide polymorphisms
SPT: SPATULA
VTC4: Inositol monophosphatase family protein
YUCCA3: Indole-3-pyruvate monooxygenase
