# Supplementary material for: GmBZL3 acts as a major BR signaling regulator through crosstalk with multiple pathways in Glycine max
Source: BMC Plant Biol. 2019 Feb 22;19:86. doi: 10.1186/s12870-019-1677-2 (PMC6387493; doi:10.1186/s12870-019-1677-2)
Supplement: Supplementary file 2 — Expression patterns of GmBZLs in different tissues and under abiotic stress conditions. (A) Expression profiles of GmBZL genes in 9 tissues. The relative expression data of 9 tissues determined by RNA-seq were obtained from Libault et al.. 2010 and used to construct the expression patterns of soybean genes. Color in the heatmaps represents RPKM values of the GmBZL genes. (B-C) Transcription levels of GmBZL genes in response to abiotic stress. The soybean V1 stage seedlings were treated with 200 mM NaCl or 100 μM ABA for 8 h. For cold treatment, seedlings were kept at 4 °C with light. For dehydration treatment, seedlings were transferred onto filter paper and dried at room temperature with 60% humidity. Relative gene expression levels are shown following normalization with actin transcript values. Error bars represent the standard error of the mean. The star (*) indicates statistically significant differences among the means (p < 0.05). (PPTX 88 kb) [file 12870_2019_1677_MOESM2_ESM.pptx]

## Slide 1
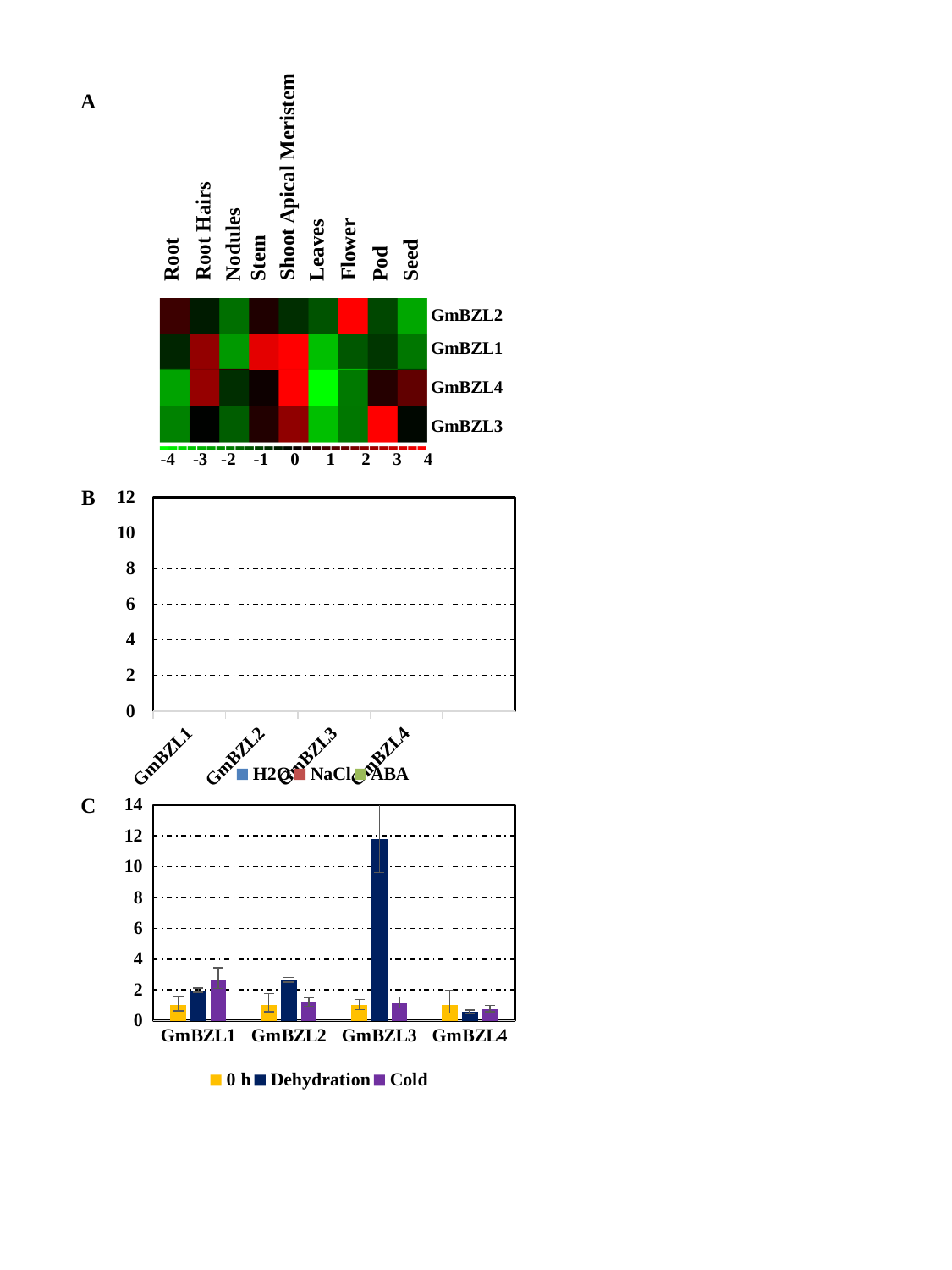

A
Shoot Apical Meristem
Root Hairs
Nodules
Leaves
Flower
Stem
Root
Seed
Pod
GmBZL2
GmBZL1
GmBZL4
GmBZL3
-4 -3 -2 -1 0 1 2 3 4
B
### Chart
| Category | H2O | NaCl | ABA |
|---|---|---|---|
| GmBZL1 | 1.0 | 1.9944141173289403 | 1.1834090501865397 |
| GmBZL2 | 1.0 | 1.6421561683629535 | 1.6460853783121643 |
| GmBZL3 | 1.0 | 11.347096902257084 | 2.2285476884158886 |
| GmBZL4 | 1.0 | 0.701539503979798 | 2.155098036896637 |C
### Chart
| Category | 0 h | Dehydration | Cold |
|---|---|---|---|
| GmBZL1 | 1.0 | 1.9728678651734868 | 2.6857161447052147 |
| GmBZL2 | 1.0 | 2.6523043503208523 | 1.1858633986856393 |
| GmBZL3 | 1.0 | 11.76153673593892 | 1.127450817082066 |
| GmBZL4 | 1.0 | 0.5603702744521303 | 0.7594548833086955 |
