# Supplementary figures and images for: GmBZL3 acts as a major BR signaling regulator through crosstalk with multiple pathways in Glycine max
Source: BMC Plant Biol. 2019 Feb 22;19:86. doi: 10.1186/s12870-019-1677-2 (PMC6387493; doi:10.1186/s12870-019-1677-2)

## Slide 1
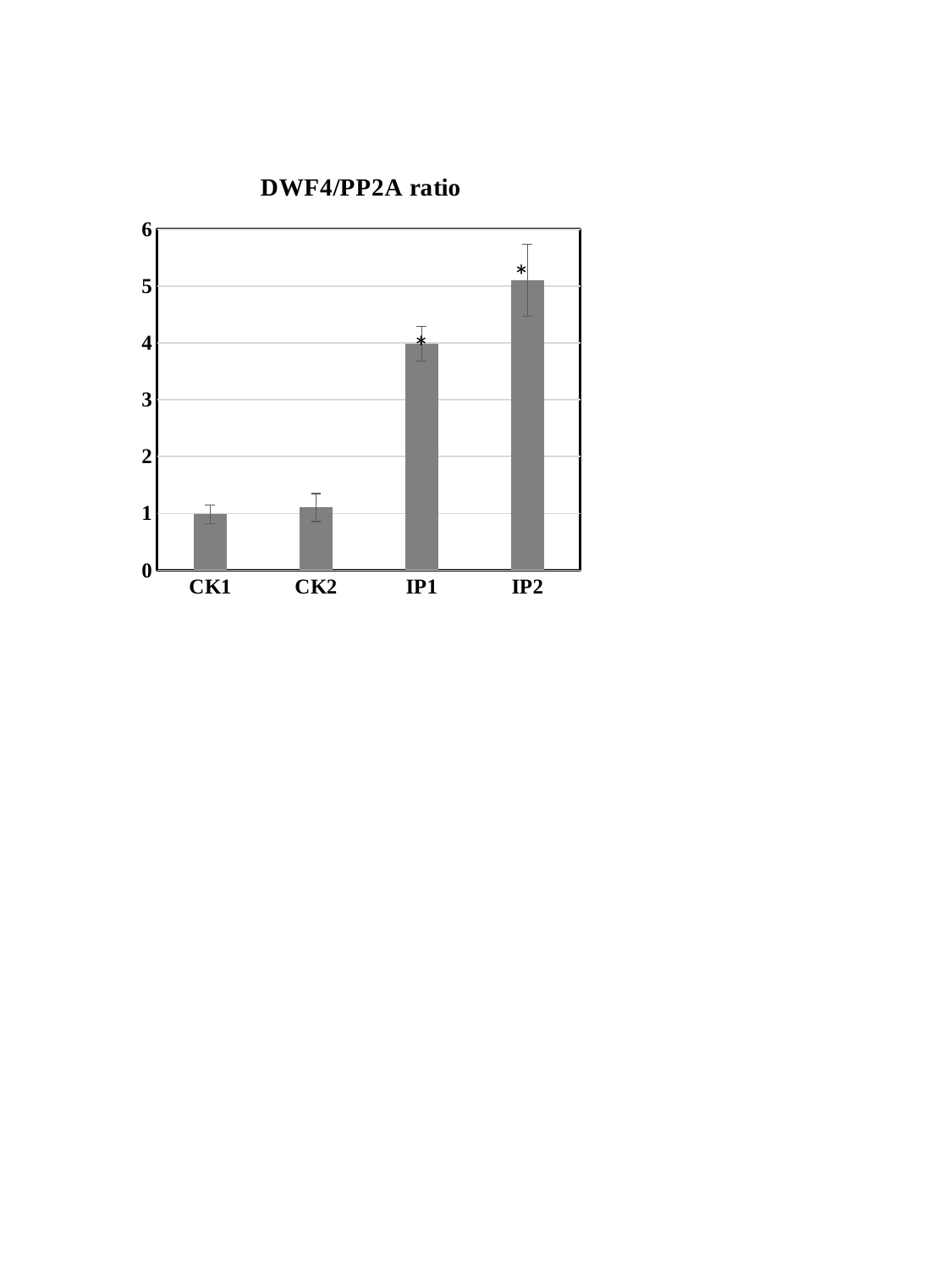

### Chart: DWF4/PP2A ratio
| Category | DWF4/PP2A |
|---|---|
| CK1 | 0.984253 |
| CK2 | 1.102589 |
| IP1 | 3.985994333333334 |
| IP2 | 5.101186166666666 |*
*

Supplement: Supplementary file 3 — DWF4 was significantly enriched in both IP biological repeat samples compared with PP2A, but no significant different of amount between DWF4 and PP2A in negative ChIP experiment. (PPTX 67 kb) [file 12870_2019_1677_MOESM3_ESM.pptx]

## Slide 1
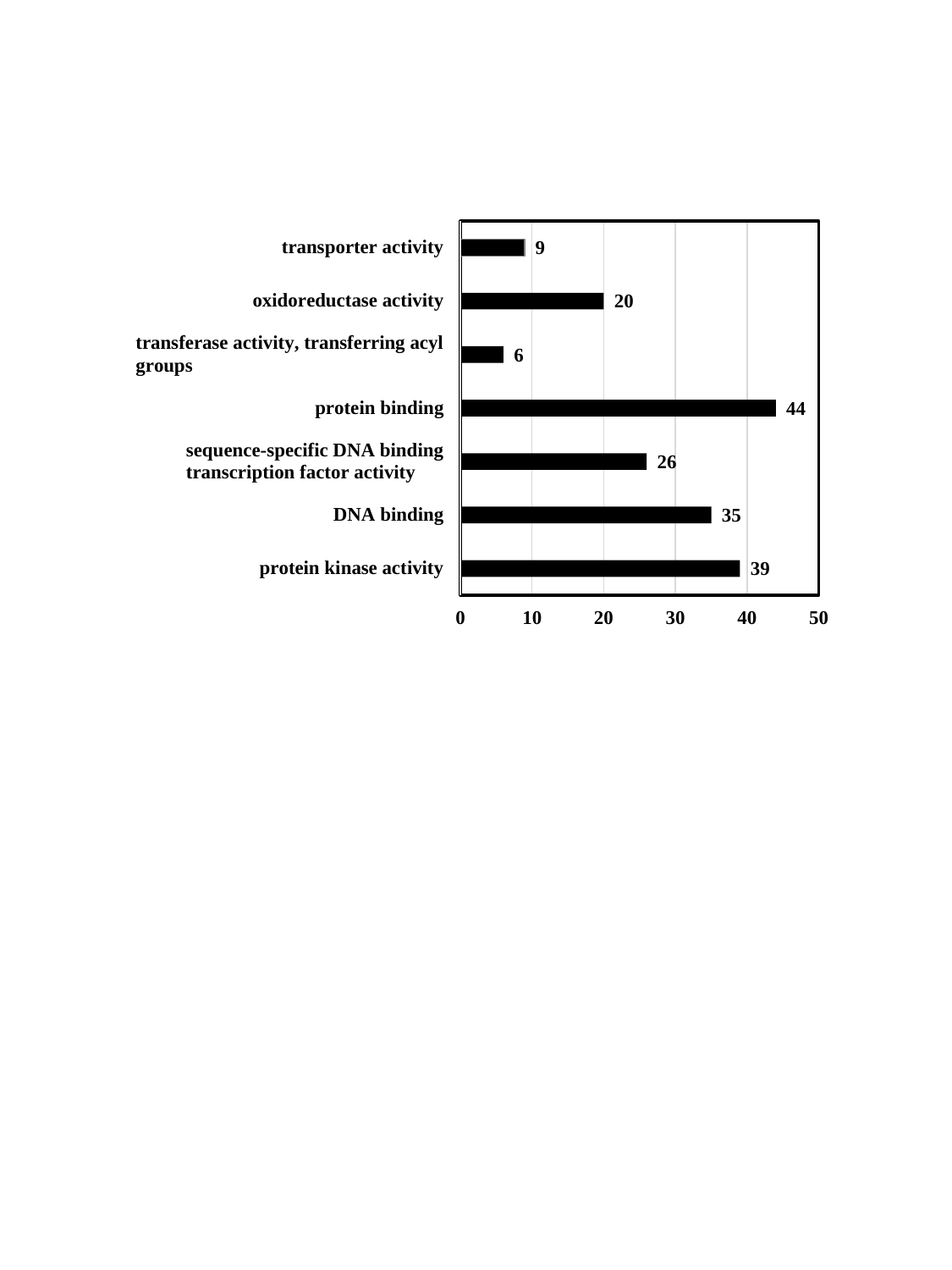

Supplement: Supplementary file 5 — Overrepresented GO categories in GmBZL3 targets. Functional classification of GmBZL3 targets indicated that seven sets of GO (molecular function) terms were significantly enriched (p values < 0.01). (PPTX 40 kb) [file 12870_2019_1677_MOESM5_ESM.pptx]
